# Alternative splicing is highly variable among *Daphnia pulex* lineages in response to acute copper exposure

**DOI:** 10.1186/s12864-020-06831-4

**Published:** 2020-06-26

**Authors:** Sneha Suresh, Teresa J. Crease, Melania E. Cristescu, Frédéric J. J. Chain

**Affiliations:** 1 Department of Biological Sciences, University of Massachusetts Lowell, Lowell, MA 01854 USA; 2 Present address: The Swire Institute of Marine Science and School of Biological Sciences, The University of Hong Kong, Pokfulam Road, Pok Fu Lam, Hong Kong SAR; 3 Department of Integrative Biology, University of Guelph, Guelph, ON N1G 2W1 Canada; 4 Department of Biology, McGill University, 1205 Docteur Penfield, Montreal, QC H3A 1B1 Canada

**Keywords:** Splicing, Copper, Metal pollution, Transcriptomics, *Daphnia pulex*, RNA-seq

## Abstract

**Background:**

Despite being one of the primary mechanisms of gene expression regulation in eukaryotes, alternative splicing is often overlooked in ecotoxicogenomic studies. The process of alternative splicing facilitates the production of multiple mRNA isoforms from a single gene thereby greatly increasing the diversity of the transcriptome and proteome. This process can be important in enabling the organism to cope with stressful conditions. Accurate identification of splice sites using RNA sequencing requires alignment to independent exonic positions within the genome, presenting bioinformatic challenges, particularly when using short read data. Although technological advances allow for the detection of splicing patterns on a genome-wide scale, very little is known about the extent of intraspecies variation in splicing patterns, particularly in response to environmental stressors. In this study, we used RNA-sequencing to study the molecular responses to acute copper exposure in three lineages of *Daphnia pulex* by focusing on the contribution of alternative splicing in addition to gene expression responses.

**Results:**

By comparing the overall gene expression and splicing patterns among all 15 copper-exposed samples and 6 controls, we identified 588 differentially expressed (DE) genes and 16 differentially spliced (DS) genes. Most of the DS genes (13) were not found to be DE, suggesting unique transcriptional regulation in response to copper that went unnoticed with conventional DE analysis. To understand the influence of genetic background on gene expression and alternative splicing responses to Cu, each of the three lineages was analyzed separately. In contrast to the overall analysis, each lineage had a higher proportion of unique DS genes than DE genes suggesting that genetic background has a larger influence on DS than on DE. Gene Ontology analysis revealed that some pathways involved in stress response were jointly regulated by DS and DE genes while others were regulated by only transcription or only splicing.

**Conclusions:**

Our findings suggest an important role for alternative splicing in shaping transcriptome diversity in response to metal exposure in *Daphnia*, highlighting the importance of integrating splicing analyses with gene expression surveys to characterize molecular pathways in evolutionary and environmental studies.

## Background

Anthropogenic activities such as mining and intensive agriculture have led to a substantial amount of heavy metal pollution in aquatic ecosystems [1]. Metal contamination poses a great threat to the overall health and survival of aquatic organisms due to the long persistence and bioaccumulation [2]. Exposure to environmental contaminants can induce genomic responses in organisms affecting reproduction and survival [3]. In aquatic invertebrates, exposure to metals such as copper has been associated with increased production of reactive oxygen species, depletion of glutathione, inhibition of oxidative phosphorylation and antioxidant systems, DNA damage and inhibition of DNA repair mechanisms [4]. Heavy metals further modulate the expression level of genes that are actively involved in protecting cells from metal-induced oxidative stress [2]. Recent genome-wide studies have provided important insights into the molecular basis of transcription in response to metals, but much less is known about the contribution of other mechanisms that regulate expression via RNA processing such as alternative splicing.

Alternative splicing is a regulatory process in eukaryotes that generates multiple messenger RNAs (mRNAs) from a single gene by selective removal or retention of exons and introns from the pre-mRNA transcript [5]. It is one of the primary mechanisms of gene expression regulation that contributes to transcriptional diversity and activity in eukaryotes [6]. An extreme example is the *Dscam* gene that can generate 38,016 potential mRNA transcripts in *Drosophila melanogaster* and over 13,000 potential mRNA isoforms in *Daphnia* via alternative splicing [7, 8]. Global analyses in eukaryotes have reported a large variation in the prevalence of alternative splicing among taxa [9–11], from about 25% of genes in *Caenorhabditis elegans* [12] and 31% of genes in *Drosophila* [13], to over 90% of genes in humans [14]. Genomic architecture has been suggested to play a role in the diversity of observed frequencies of different types of alternative splicing [15]. The main types of alternative splicing events include exon skipping (ES), intron retention (IR), alternative 5′ splice site (A5SS), alternative 3′ splice site (A3SS) and mutually exclusive exons (MXE) [16]. Exon skipping is the most common type of alternative splicing in animals, while intron retention events occur at high levels in plants, as well as most fungi and protists [11, 17]. Exon skipping, the presence of MXE, A3SS and A5SS within the exons lead to addition or removal of functional domains or changes in the amino or carboxy terminus of the protein product thereby affecting its activity, localization, stability and function. Intron retention events usually lead to premature stop codons within the transcripts, which results in the formation of a truncated protein or in most cases degradation of the transcript thereby regulating the amount of functional transcript present in the cell [18].

Identification of alternative splicing events from RNA-seq data involves mapping reads to a reference genome to identify splice junctions and counting the number of reads aligning to particular exons and splice sites. Percent Spliced In (PSI) is a widely used metric for quantifying alternative splicing events and detecting differential splicing between conditions. It represents the percentage of transcripts including a particular exon or splice site and is calculated from the read counts [19]. Unlike gene expression analysis where a read falling anywhere along the gene will count towards expression, identification of a splicing event requires the read to span the splice junction and hence detection of differential splicing events could potentially be biased towards more abundant transcripts [20]. Additionally, abundance of the final mRNA transcript is dependent on several factors such as cell type, developmental stage, disease condition, the presence of intronic and exonic enhancers and silencers, the expression of various splicing factors, and can change in response to external stimuli and cellular stress [21]. Identification of intron retention events could be particularly challenging as it can be difficult to distinguish true intron retention events from those arising from incompletely processed transcripts [22]. However, several computational tools have been developed for analysing alternative splicing events from RNA-seq data. While some tools like DEXSeq [23] rely on reads assigned to exons, others like JunctionSeq [24] and rMATS [25] use reads aligned to both exons and splice junctions and can therefore identify differential splicing events even when the exon expression level is consistent across different conditions [26].

Several studies have reported that alternative splicing plays an important role in abiotic stress tolerance in plants and mammals [27–30], but whether alternative splicing is a common response to stress in aquatic invertebrates is not well understood. Analyses of whole transcriptomes of plants have shown that alternative splicing regulates the expression level of genes involved in stress response pathways and genes encoding the various components of a spliceosome, which is an RNA-protein complex that directs splicing of pre-mRNA transcripts [31]. Recent studies on plants have also shown that abiotic stresses such as exposure to high temperatures, high salinity or treatment with the plant hormone abscisic acid alters the alternative splicing patterns of several genes and promotes the use of non-canonical splice sites, thereby increasing the transcriptome diversity in adverse environmental conditions [30]. Similar studies on nematodes and insects suggest that regulation of alternative splicing events is a key mechanism in mediating response to stressors, reporting alternative splicing mediated regulation of transcriptional activity in response to heat/cold stress [32–35]. In addition, a genome-wide analysis of alternative splicing events in the Pacific oyster, *Crassostrea gigas* in response to abiotic stressors reported that 16% of the oyster protein coding genes undergo alternative splicing and these genes are enriched in functions related to cellular metabolism, cell signaling, and post translational protein modifications [36]. There is a lack of similar analyses of the relative contribution of alternative splicing in regulating gene expression in most organisms, including *Daphnia pulex*, a well-established model organism for ecological genomics. Differential splicing analysis has the potential to identify functional diversity that is missed by differential gene expression analysis alone, and hence can complement differential gene expression in understanding the genes and molecular pathways involved in stress response. Altering the splicing patterns is a major mechanism that can regulate the levels of gene expression and inhibit protein synthesis by introducing premature stop codons in the mRNA resulting in their degradation by the mRNA surveillance system [37, 38]. Furthermore, individual variation in alternative splicing patterns have been shown to alter the phenotypic response to stress in various organisms, suggesting that splicing can vary between genotypes [39]. Although alternative splicing seems to play an important role in stress response mechanisms, more studies are needed to identify the extent of splicing upon exposure to stress as well as the variability within species and how it complements the transcriptional response.

The micro-crustacean *Daphnia pulex* is among the most common species of water flea inhabiting lakes and ponds throughout the world. It was the first crustacean to have its genome sequenced and is widely used as a model organism in environmental toxicity studies [40]. *Daphnia* has the ability to develop distinct alternative phenotypes in response to environmental cues and has been considered to have an ecologically responsive genome [41]. Based on the newest genome assembly, *D. pulex* has a compact genome of 156 Mb consisting of 18,440 genes with relatively small introns and small intergenic spaces [42]. Previous work has identified that 51% of *D. pulex* genes and 60% of *Daphnia magna* genes undergo alternative splicing [43]. *Daphnia* occur in diverse environments across a wide range of ecological conditions and the populations have a high degree of genetic variation [44, 45]. Genetic divergence between *Daphnia* populations could result in varied phenotypic responses to stressors [46]. Consequently, the effects of stressors on monoclonal populations cannot be extrapolated to the species level as genetically diverse populations will differ in tolerance and response to stress [47]. Previous studies have reported differences among *Daphnia* clones in tolerance and response to various natural and anthropogenic stressors [46–49], but there is a lack of similar studies in response to metal stress. Heavy metal concentrations in the environment continue to be a concern with ongoing industrial activities [50], and copper is one of the most common pollutants that is toxic at high concentrations [51]. Exposure to sub-lethal concentrations of copper is known to significantly impair reproductive output in *D. pulex* [52]. Our recent study found that most differentially expressed genes between copper-exposed *Daphnia* and controls were shared among genetic lineages, but each lineage had a few unique genes that changed in expression under copper exposure [53]. Thus, while stress response mechanisms may be largely similar among the members of a species, individual populations may adopt different mechanisms to adapt to environmental perturbations. Investigating the genetic basis of differential gene expression and splicing among *Daphnia* clones can help distinguish common stress response pathways from lineage-specific responses to metal exposure.

In this study, we integrate an analysis of differential gene expression and differential splicing to identify the role of alternative splicing in mediating response to metal-induced acute stress in *Daphnia.* We perform these analyses using our previously published RNA-seq dataset on lineages that originate from three natural populations, which was used to determine the extent of similar responses to Cu among lineages [53]. Here, we add new analyses to determine whether differentially spliced genes have similar functional enrichment distributions and regulate similar biological processes as differentially expressed genes, and we also use a new reference genome assembly with refined gene annotations [42]. This work advances our understanding of the biological significance of alternative splicing events in *Daphnia* and its impact in shaping the transcriptome diversity in response to metal exposure.

## Results

### Differential gene expression in response to acute copper exposure

**Fig. 1:**
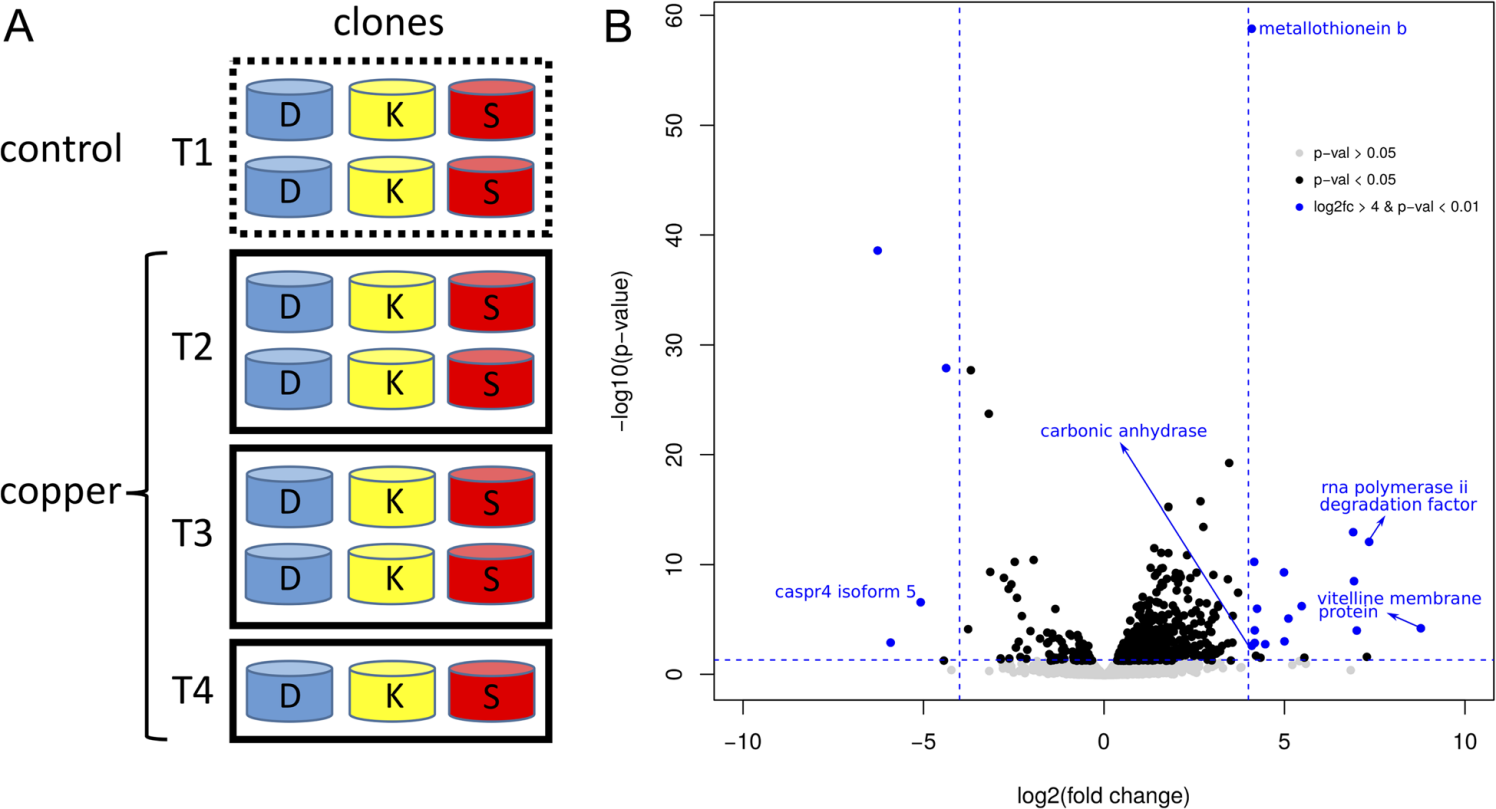
**a**: Experimental set-up for investigating the molecular responses of *D. pulex* to acute Cu exposure. Individuals from three different clonal lineages (D, K, S) were placed in individual tubes in four separate tanks. **b**: Volcano plot showing gene expression fold change differences between copper-exposed and control samples. The average log2 fold change in gene expression is on the x-axis (positive values are upregulated in copper-exposed samples), and the average negative log10 of FDR-corrected *p*-values are on the y-axis. Non-differentially expressed genes are in grey (corrected *p*-value > 0.05), differentially expressed (DE) genes (corrected *p*-value < 0.05) are in black and DE genes with corrected *p*-value < 0.01 and log2 fold change > 4 are in blue. The five labelled genes correspond to the annotated DE genes that are strongly responsive to acute Cu exposure in Table 1. The blue dotted lines indicate the cut-off values for the log2fold change and FDR-corrected *p*-value for the genes identified to be strongly responsive to acute Cu exposure.

A total of 21 *Daphnia* RNA-seq samples were used to determine transcriptional responses to Cu exposure (Fig. 1a). Across all samples, there was an average of 16,151 expressed genes (Table S1), and an average read depth of 498 per gene and 9.9 per coding bp*.* A total of 588 differentially expressed (DE) genes were identified between all 15 samples exposed to acute Cu stress versus all 6 control samples (FDR corrected *p*-value < 0.05). DE genes with known functional annotations accounted for 64% (377/588) of all DE genes, similar to the genome-wide proportion (63%). Five hundred genes were upregulated (log2fc > 0) in response to acute Cu exposure and 88 genes were downregulated (Fig. 1b; Table S2). Of the DE genes, 20 genes had an FDR corrected *p*-value < 0.01 and an absolute log2 fold change > 4, which we identified as having the most extreme expression differences between copper and controls (Fig. 1b). Five of these 20 DE genes are annotated and characterized: *metallothionein b (mtb)*, *alpha-carbonic anhydrase (aca1)*, *vitelline membrane outer layer protein 1 (vmo1), rna polymerase ii degradation factor 1 (def1), and cell recognition protein (caspr4) isoform 5*. Six of these 20 DE genes were also reported as DE in our previous study [53] (Table 1), which used the same transcriptomic dataset but the *D. pulex* genome published in 2011 [41]. We conducted a BLAST analysis to compare the DE genes identified in this study against the genes from the 2011 genome assembly. Although our previous analyses reported in Chain et al. [53] reported three times fewer DE genes than we did in this new analysis (206 genes out of an average of 17,128 expressed genes), 142 genes out of the 206 genes (69%) were DE in both analyses, and only seven genes with reciprocal blast hits were not DE in the current study. A total of 40 genes did not have any reciprocal blast hits, probably because there are multiple duplicate genes in the 2011 genome (Tables S3, S4), as suggested by Ye et al. [42].

**Table 1:** Differentially expressed (DE) genes that are strongly responsive to acute Cu exposure. Results are based on the global analysis combining all 6 controls with all 15 Cu exposed samples. Genes that were also reported to be strongly responsive to Cu exposure in a previous study by Chain et al., [53] using the 2011 draft genome are indicated by an asterisk (*)

Gene_ID	Description	blast hit with the 2011 genome annotation	log2 fold change		FDR corrected ***p***-value	
			edgeR	DESeq2	edgeR	DESeq2
gene8176	hypothetical protein DAPPUDRAFT_104167	104167*	−6.24	−6.32	5.82E-44	4.45E-39
gene17246	*cell recognition protein caspr4 isoform 5*	109980*	−5.05	−5.13	2.07E-07	2.51E-07
gene8175	hypothetical protein DAPPUDRAFT_225009	225009*	−4.35	−4.41	4.49E-32	2.17E-28
gene5445	hypothetical protein DAPPUDRAFT_313428	313428*	4.98	4.97	8.54E-10	1.49E-17
gene3837	hypothetical protein DAPPUDRAFT_222529	222529*	6.92	6.91	5.40E-09	1.62E-20
gene16955	*rna polymerase ii degradation factor 1-like*	101472*	7.25	7.41	3.66E-16	1.41E-12
gene7919	hypothetical protein DAPPUDRAFT_324898	---NA---	−5.73	−6.11	3.47E-04	1.83E-03
gene7984	*metallothionein b*	290503	4.09	4.07	2.83E-59	5.07E-132
gene6693	*alpha-carbonic anhydrase*	---NA---	4.08	4.08	4.18E-03	3.41E-05
gene7509	hypothetical protein DAPPUDRAFT_335675	---NA---	4.16	4.14	9.39E-11	3.37E-18
gene6523	---NA---	---NA---	4.03	4.30	1.05E-06	1.65E-04
gene3259	hypothetical protein DAPPUDRAFT_211890	---NA---	4.17	4.16	2.33E-03	1.05E-05
gene17951	hypothetical protein DAPPUDRAFT_313929	---NA---	4.23	4.23	1.76E-06	3.88E-11
gene8356	hypothetical protein DAPPUDRAFT_106354	---NA---	4.44	4.47	3.05E-03	1.97E-05
gene5796	hypothetical protein DAPPUDRAFT_229695	---NA---	4.98	4.99	1.67E-03	4.49E-06
gene9671	hypothetical protein DAPPUDRAFT_333097	333097	5.10	5.10	1.39E-05	1.12E-10
gene12057	hypothetical protein DAPPUDRAFT_222523	---NA---	5.39	5.54	3.92E-08	9.87E-07
gene5697	hypothetical protein DAPPUDRAFT_337500	337500	6.75	7.03	1.06E-30	1.85E-13
gene2964	hypothetical protein DAPPUDRAFT_244715	---NA---	7.28	6.70	1.60E-04	1.30E-05
gene1954	*vitelline membrane outer layer protein 1-like*	---NA---	8.68	8.85	1.07E-04	1.30E-07

### Identification of alternative splicing events in *Daphnia*

Across the three clonal lineages, a total of 6630 of the 17,761 expressed genes were identified to have alternative transcripts, accounting for ~ 37% of the *D. pulex* genes (Table S1)*.* Specifically, 4820, 4738 and 4721 alternatively spliced (AS) genes were identified in Clones K, S and D, respectively. This is slightly more than the percentage of alternatively spliced genes in other species such as *C. elegans* (25%) [12], *C. gigas* (16%) [36] and *D. melanogaster* (31%) [13], but less than a previous estimate of 51% [43]. Five AS types were inferred: exon skipping (ES), intron retention (IR), alternative 5′ splice site (A5SS), alternative 3′ splice site (A3SS) and mutually exclusive exons (MXE). The distribution of AS types was similar across the lineages, with A3SS being the most abundant (52–59%), followed by A5SS (45–50%), IR (22–23%), ES (18–19%) and MXE (2%) (Table 2).

**Table 2:** Distribution of alternative splicing (AS) types among the three clonal lineages

AS Type	Clone D		Clone S		Clone K		Total
	No. of genes	percentage	No. of genes	percentage	No. of genes	percentage	
A3SS	2477	52.5	2808	59.3	2669	55.4	4174
A5SS	2398	50.8	2143	45.2	2303	47.8	3796
MXE	104	2.2	111	2.3	115	2.4	129
ES	906	19.2	879	18.5	940	19.5	1181
IR	1058	22.4	1120	23.6	1076	22.3	1666

### Differential splicing events in response to acute copper exposure

Comparisons of the alternative splicing events between all 15 copper-exposed samples with all 6 control samples identified 16 significantly differentially spliced (DS) genes (FDR corrected *p*-value < 0.05) with a difference in exon inclusion level greater than 20% (Table 3). Functional annotations of these genes included ion binding, DNA binding, transcription regulation, transmembrane transport, signal transduction, metabolism, protein ubiquitination, serine-type endopeptidase activity and proteolysis. The alternatively spliced exons in 5 of these DS genes involve conserved domain superfamily clusters related to functions that promote the insertion of copper into cytochrome c oxidases (COX16), cellular detoxification (GST-C family), and anion translocation across membranes (ArsB NhaD permease; Table S5). Among the DS genes, ES was the most abundant splicing type (6 genes) followed by A3SS (5 genes), A5SS (4 genes), MXE (2 genes) and IR (1 gene) (Table 3; Table S5). Three DS genes – *glutathione s-transferase (gst), alpha-aspartyl dipeptidase (pepe),* and *transmembrane protein 189 (tmem189)* – were also found to be differentially expressed.

**Table 3:** Genes differentially spliced in response to acute Cu exposure from the global analysis combining all 6 controls with all 15 Cu exposed samples. Δψ is the absolute value of the difference in exon/intron inclusion levels between controls and Cu exposed samples

Gene ID	Description	GO Term	AS type	Spliced region	|Δψ|
gene15738	*coatomer subunit gamma*	binding	A3SS	Exon 3	0.255
gene7414	*lethal malignant brain tumor-like protein 3 isoform ×2*	transcription regulation; zinc ion binding	A3SS	Exon 7	0.342
gene16738	*sialin isoform ×2*	transmembrane transport	A3SS	Exon 8	0.291
gene4489	*sprouty- evh1 domain-containing protein partial*	signal transduction	A3SS	Exon 4	0.293
gene8923	hypothetical protein DAPPUDRAFT_267459	metabolism	A3SS	Exon 3	0.281
gene7598	hypothetical protein DAPPUDRAFT_309480	single-stranded DNA binding	A5SS	Exon 1	0.302
gene9075	*nucleoside diphosphate kinase 7*	metabolism	A5SS	Exon 1	0.211
gene9075	*nucleoside diphosphate kinase 7*	metabolism	ES	Exon 1 partial	0.244
gene11416	*protein grainyhead isoform ×1*	NA	A5SS	Exon 12	0.346
gene5664	*type i procollagen alpha 1 chain*	NA	A5SS	Exon 4	0.202
gene1314	*glutathione s-transferase*	protein binding	MXE	Exon 4 and 9; Exon 5 and 10	0.267
gene8790	*i’m not dead yet*	transmembrane transport	MXE	Exon 11 and 12	0.301
gene8790	*i’m not dead yet*	transmembrane transport	ES	Exon 12	0.324
gene14576	*pyruvate kinase-like isoform x*	ion binding; metabolism	ES	Exon 9	0.204
gene7153	*tgf-beta-activated kinase 1 and map 3 k7-binding protein 2*	zinc ion binding	ES	5′ UTR	0.278
gene377	*alpha-aspartyl dipeptidase*	serine-type peptidase activity; proteolysis	ES	Exon 2	0.231
gene10569	*endophilin-a isoform ×1*	protein binding; endocytosis	ES	Exon 8	0.213
gene15982	*transmembrane protein 189*	protein ubiquitination	IR	Intron 3	0.286

### Lineage-specific patterns of gene expression and splicing

**Fig. 2:**
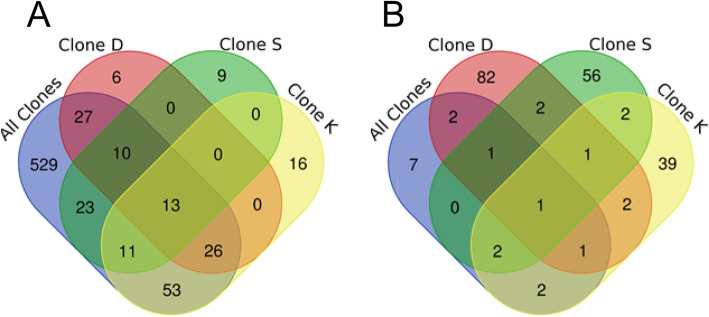
Venn diagram showing the overlap of differentially expressed and differentially spliced genes. Venn diagrams show the overlap of genes that display (**a**) differential expression (DE) or (**b**) differential splicing (DS) in the global analysis of all clones and the three separate analyses of clonal lineages. DE and DS analysis was carried out by grouping all clonal populations together (6 control samples vs 15 copper samples) or separately for each individual clone (2 control samples vs 5 copper samples)

To investigate the influence of genetic background on the molecular response to acute copper exposure, the gene expression and splicing patterns were compared between controls (*n* = 2) and copper-exposed samples (*n* = 5) of each clonal lineage. A total of 82 DE genes were identified in Clone D, 119 DE genes in Clone K and 66 DE genes in Clone S (FDR corrected *p*-value < 0.05; Table S6). Of these, 6 DE genes were unique to Clone D, 16 were unique to Clone K and 9 were unique to Clone S. Over 80% of the DE genes from clone-specific analyses overlapped with DE genes from the global analysis that combined all clonal lineages, suggesting that responses to acute Cu exposure are generally shared among all clones (Fig. 2a). This is consistent with our previous results reported in Chain et al. [53] using the same dataset but a different reference assembly. *Metallothionein b (mtb)*, *lipase 3 (lip3)*, *rna polymerase ii degradation factor 1 (def1), urea transporter 1-like (slc14A1), multiple inositol polyphosphate phosphatase 1 (minpp1)* and eight other genes with unknown functions were commonly differentially expressed in the clone-specific analysis and in the analysis combining all clonal lineages (Table S7). This suggests that these genes consistently play a significant role in mediating transcriptional response to acute Cu exposure in *Daphnia* regardless of genetic background.

**Fig. 3:**
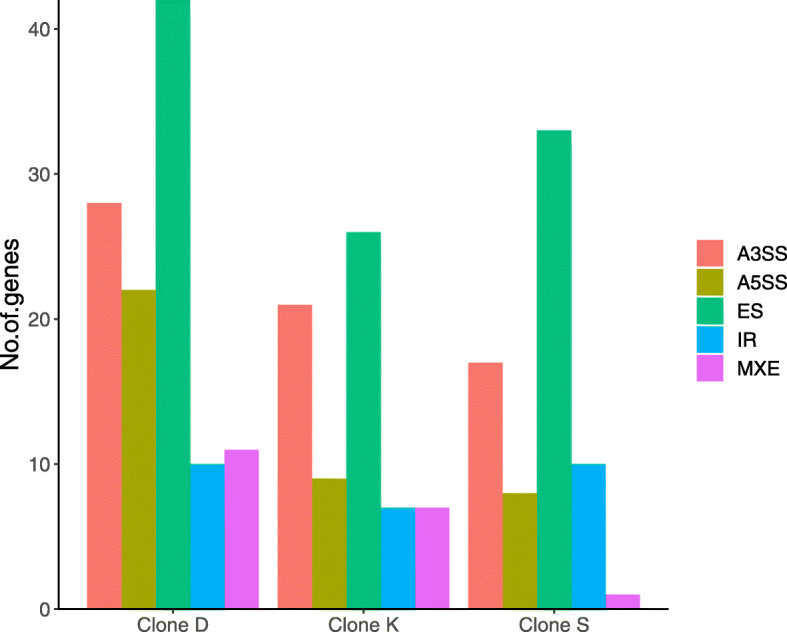
Number of differentially spliced (DS) genes according to splicing type. The DS splicing type is shown for Clones D, S and K in response to acute copper exposure. A3SS – alternate 3 prime splice site; A5SS – alternate 5 prime splice site; MXE – mutually exclusive exons; ES – exon skipping; IR – intron retention

**Fig. 4:**
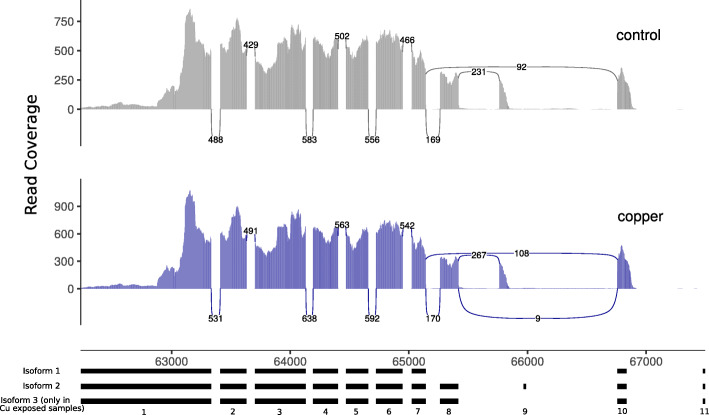
Differential splicing of gene14576 (pyruvate kinase-like isoform x). The x-axis represents the exons and the y-axis represents the read coverage. The curved lines indicate splicing. Skipping of exon 9 is observed only in individuals exposed to copper (supported by an average of 9 reads across all 15 Cu exposed samples). Note that the y-axis scale is different in copper and control

In contrast to DE genes, there was very little overlap in DS genes among lineages: a total of 68 DS genes were identified in Clone S, 101 DS genes in Clone D, and 65 DS genes in Clone K (FDR corrected *p*-value < 0.05 and a difference in exon inclusion level greater than 20%), out of which 56 were unique to Clone S, 82 were unique to Clone D and 39 were unique to Clone K (Fig. 2b). The most common differential splicing type observed in all clones was exon skipping followed by use of alternative 3′ splice site and use of alternative 5′ splice site (Fig. 3; Tables S8, S9, S10). One gene (gene14576; *pyruvate kinase-like (pk) isoform x*) was found to be differentially spliced in all comparisons (including in each clone separately), suggesting that it plays an important role in post-transcriptional Cu stress response in all clones regardless of the genetic background. Exon 9 of this gene was skipped in samples exposed to Cu (Fig. 4), but the effect of this alternate transcript on protein function is unknown and no conserved domains were found overlapping this exon (Table S5)*.* Interestingly, Clone K, which came from a copper-contaminated lake, had the least number of DS genes but the highest number of DE genes.

### Gene ontology (GO) enrichment analysis of DE and DS genes

A total of 45 Gene Ontology (GO) terms mostly belonging to 11 major functional categories were enriched among the upregulated DE genes, and 20 GO terms belonging to 10 major functional categories were enriched among the downregulated DE genes (weighted *p*-value < 0.05; Table S11). After applying an FDR correction, only five GO terms remained enriched among upregulated genes - proteolysis, serine-type endopeptidase activity, chitin binding, chitin metabolic process and metallocarboxypeptidase activity; all these GO terms were also reported to be significantly enriched among the upregulated DE genes in our previous analyses [53]. Only one functional category was enriched among the downregulated genes after FDR correction, extracellular matrix structural constituent, which was also identified to be enriched in our previous analyses [53]. Before any FDR correction, enrichment analysis of DE genes from clone-specific analyses identified numerous enriched GO terms that mostly overlap with the global analysis, including the five functions significant after FDR correction (Table S12). Because the topGO enrichment analysis algorithms already account for gene ontology topology, the topGO authors do not necessarily recommend the conservative FDR correction [54].

**Fig. 5:**
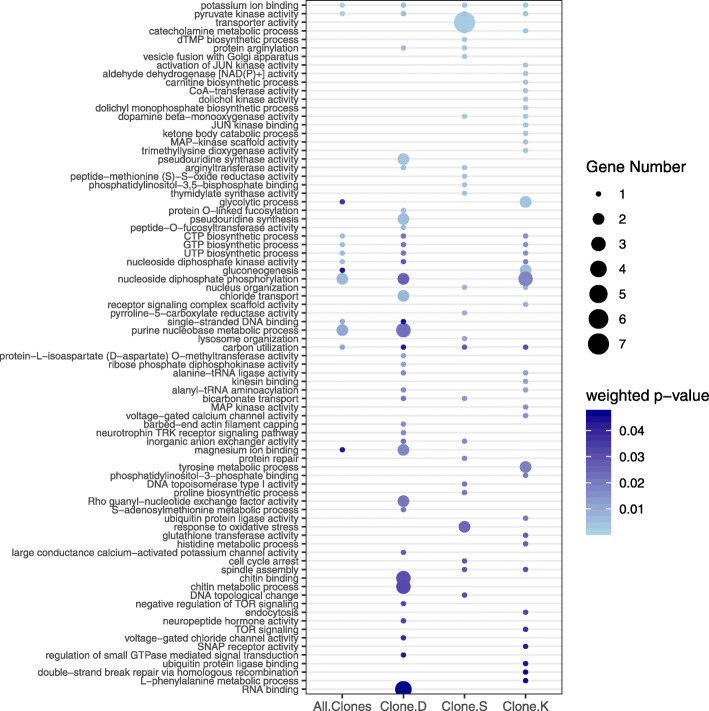
Functional enrichment of differentially spliced genes. Distribution of enriched gene ontology (GO) terms for differentially spliced genes from the global analysis of all clones grouped together as well as from each individual clone analysis. The size of the circles is proportional to the number of observed genes within each GO category and the shade of the circles is proportional to the significance (measured in terms of the weighted *p*-value reported by topGO)

Among the DS genes, a total of 16 GO terms belonging broadly to the five functional categories of metabolism, binding, catalytic activity, carbon utilization and transport were enriched (weighted *p*-value < 0.05) when all clones are combined (6 controls and 15 Cu-exposed samples; Table S13). These same major categories plus a few more were also enriched among the clones; Clones D, S and K had 36 (7 functional categories), 25 (8 functional categories) and 38 (9 functional categories) GO terms enriched, respectively (weighted *p*-value < 0.05; Fig. 5; Table S14).

### Comparison between transcriptional and splicing responses to acute Cu exposure in *Daphnia pulex*

We found a much higher number of genes undergoing differential expression than differential splicing in response to acute Cu exposure from the global comparison of all clones together: on average, 3.6% of all genes expressed across the 21 samples were differentially expressed compared to differential splicing of only 0.7% of the genes with alternative transcripts (0.1% of the expressed genes). However, from the clone-specific analyses, each independent clone has a higher number of unique DS genes than unique DE genes, with Clone D having the highest proportion of unique DS vs DE genes (82/6) followed by Clone S (56/9) and Clone K (39/16). This suggests that the splicing patterns induced by Cu are more variable between genetic lineages than gene expression levels. The functional categories of metabolism, catalytic activity, binding and transport were commonly enriched among the DE and DS genes in both the global analyses and all three clone-specific analyses suggesting that these pathways are regulated both at the level of transcription and splicing in response to Cu. Certain functions such as signal transduction, stress response and cellular component organization were enriched among the DE but not the DS genes in the global analyses, but were enriched among the DS genes in the clone-specific analyses. For example, signal transduction is enriched among the DE genes but only enriched among the DS genes in Clones D and K. Similarly, stress response is enriched among the DE genes but only among the DS genes in Clones S and K. Cellular component analysis is enriched among the DS genes in all the three clones, but it is only enriched among the DE genes in Clone K. This suggests that some key pathways involved in response to metal exposure are jointly regulated by gene expression and splicing, but genetic variation between the clonal lineages also influences the molecular responses observed.

## Discussion

Alternative splicing is an important regulatory mechanism that increases transcriptome and proteome diversity and could potentially play an important role in mediating response to stress in addition to gene expression changes. Most studies investigating the molecular mechanisms of response to environmental stressors have focused on gene expression responses without considering the role of alternative splicing. To our knowledge, this is the first study investigating genome wide changes in alternative splicing patterns in *D. pulex.* By comparing both the gene expression and splicing patterns in response to acute copper exposure in three *D. pulex* lineages, we provide evidence that alternative splicing is prevalent and highly variable among lineages in response to acute Cu stress.

To investigate the molecular responses to acute copper exposure, we performed new analyses on our previously published dataset on gene expression in *D. pulex* [53]. Our previous study focused on gene expression differences rather than differential splicing response to acute Cu exposure, and also utilized an older genome reference that is reported to have assembly errors leading to an overestimation of gene number [42]. However, consistent with our previous study, we found that genes involved in metabolism, digestion, immune response, ion binding and transport, signal transduction, chitin binding, exoskeletal protein metabolism and chitinase activity, oxidative stress response and metal detoxification were significantly differentially expressed [53, 55, 56]. Most of the DE genes identified in our previous study [53] were also detected in our current study, and the enriched functional categories were almost identical. It is however notable that our analysis using the most recent genome assembly detected almost three times as many DE genes, suggesting an improved ability to detect differential gene expression when a better-assembled genome is used. Out of the 20 genes that were found to have a 16-fold change in expression in copper-exposed samples compared to controls, only 6 were also reported to be strongly responsive to Cu exposure in our previous study using the older reference genome assembly [53]. Follow-up functional studies are needed to confirm this and to understand their potential role in mediating response to Cu stress in *Daphnia.*

### Differential splicing response to acute Cu exposure

Over 30% of the expressed genes across all 21 samples underwent alternative splicing, and 16 genes were differentially spliced in response to copper when comparing all 15 copper-exposed samples to the 6 controls. The gene *pyruvate kinase-like (pk) isoform x* (gene14576) was identified to be significantly differentially spliced both in the global analysis and in all three clones individually. The enzyme pyruvate kinase (PK) catalyzes the final reaction of glycolysis and is known to have 4 isoforms in mammals (M1, M2, R and L). The M1 and M2 isoforms, which are derived from alternative splicing of the same gene (*pyruvate kinase M - pkm*), are identical except for a 160 bp section within the coding region [57]. The activity of PK is known to be inhibited by oxidative stress in species ranging from bacteria to humans. In *S. cerevisiae*, low PK activity resulting from oxidative stress has been associated with increased respiration which suppresses the production of reactive oxygen species [58]. In *D. pulex*, this gene consists of 11 exons and has two mRNA isoforms in both controls and Cu-exposed samples. One of the isoforms consists of exons 1 to 9 while the other isoform consists of exons 1–7 and 10–11. Here we found differential splicing where exon 9 was skipped only in samples exposed to Cu, suggesting the presence of a shorter transcript isoform of this gene expressed only in response to Cu exposure (Fig. 4). Further investigation of the functional role of this additional mRNA lacking exon 9 can help understand whether it has a role in mitigating Cu-induced oxidative stress in *Daphnia.* Additionally, there is one exon in the two genes *nucleoside diphosphate kinase 7* (*ndk7*; gene 9075) and *i’m not dead yet* (*indy*; gene 8970) that is involved in multiple splicing events. While it is possible for one gene to have multiple transcript isoforms, further investigation is required to understand the exact splicing mechanism of these genes and the putative functional role of the alternative transcripts in relation to Cu stress.

### Clone-specific patterns of differential splicing

To investigate whether different genotypes of *D. pulex* vary in their response to acute copper exposure, we analyzed patterns of gene expression and splicing separately in each clone. Interclonal variation in heavy metal sensitivity has been shown in plants [59, 60] and previous studies on *Daphnia* have reported that molecular response to heavy metals varied across genotypes [49, 53, 61]. Gene expression response to acute Cu stress was more similar among the three clones compared to splicing. The three clones have on average 7.4 times more unique DS genes than unique DE genes, suggesting that genetic background has a larger effect on differential splicing than on differential expression. These intraspecific differences could reflect diverse pathways used to cope with Cu stress, but could also be the result of perturbations of the splicing machinery induced by Cu [62]. The relative abundance of a specific transcript isoform of a gene depends on three factors: (a) the rate of transcription of the gene, (b) the rate of splicing of the primary transcript to produce the specific transcript isoform, and (c) a combination of both [63]. Therefore, changes in the splicing ratios of exons/introns can result in changes in the transcript abundances even without any change in overall gene expression. Alternative splicing plays an important role in expanding proteome diversity, and higher phenotypic complexity of eukaryotes has been attributed to a high frequency of AS events. Previous studies exploring the extent of gene expression and splicing conservation across vertebrate species have reported that although alternative splicing is conserved in a subset of orthologous exons and in certain tissues such as brain and heart, alternative splicing diverges more rapidly between species than gene expression [64, 65]. Species-specific differences in alternative splicing patterns were also shown to have a more profound effect on pharyngeal jaw diversification in Lake Tanganyika cichlids than differences in gene expression, indicating that alternative splicing plays an important role in the evolution of distinct morphologies in cichlid adaptive radiation [66]. This highlights that at least among vertebrates, alternative splicing is a potentially powerful mechanism shaping species-specific differences. Differences in alternative splicing patterns between species are possibly caused by changes in the cis-regulatory elements (such as splicing enhancers or silencers) or trans-acting factors [65]. The three clonal lineages used in this study have had different Cu exposure histories, which might have led to differential selection of genotypes related to Cu tolerance in their originating natural populations [53] and differences in cis-regulatory elements or trans-acting factors. Thus, differences in splicing patterns among the clones could possibly be linked to their historical exposure to Cu and differences in Cu sensitivity.

Genes related to metabolism, transport, binding, catalytic activity, oxidative stress response, metal homeostasis and detoxification were commonly DE and DS across all three clonal lineages. Previous studies on *Daphnia* investigating transcriptional response to heavy metal toxicity have reported differential expression of genes involved in these pathways suggesting that these are key pathways associated with response to metal toxicity [53, 55, 67]. The fact that we find the genes involved in these pathways to also be DS indicates that they are regulated via transcription and splicing. In contrast, certain functional pathways were only enriched either among the DE or DS genes. The genes related to exoskeletal protein metabolism and immune response were only enriched among the DE genes whereas the genes related to post-translational modification (PTM) were only enriched among the DS genes. In addition to regulating the expression of genes in response to stressors, PTM of existing proteins can help in mitigating the effects of the stressor and restoring cellular homeostasis especially after transient exposure [68]. Exposure of yeast to increased levels of Cu results in proteolysis of the copper transport gene *ctr1*, and exposure to Zn causes ubiquitination of the Zn transporter gene *zrt1,* thereby preventing further uptake of Cu or Zn respectively [69, 70]. Thus, post-translational control provides a fast-acting alternative approach to mitigating Cu toxicity. To our knowledge, none of the previous studies on *Daphnia* investigating gene expression response to metal toxicity have reported differential expression of genes involved in the PTM process suggesting that the involvement of these genes in response to Cu stress occurs solely via splicing.

## Conclusion

Our findings suggest that genetic variation has a large impact on molecular responses to acute Cu exposure in *D. pulex*. A limitation of our study is that it included only two control replicates per clonal lineage, and hence future studies with larger sample sizes investigating the influence of interclonal variation on gene expression response to metal stress would be needed to validate our findings. In addition, it is possible that we underestimated splicing events of lowly expressed genes or those with few reads spanning splice junctions. While we found certain functions involved in Cu stress response to be common across all clones, there was variation in Cu stress-response pathways among the clones. As the lineages in our study varied in their prior exposure to sub-lethal levels of Cu, acclimation or adaptation to an environment with high Cu levels in the wild could have influenced the level of variation found in the splicing results, if this effect persists for many generations after culturing in the lab in the absence of Cu. Alternatively, high levels of variation could be driven by perturbations of the splicing machinery by copper stress. By comparing the transcriptional and splicing response to acute Cu exposure, we conclude that interclonal variation can have a substantial influence on splicing and argues for an increase in attention to alternative splicing in toxicogenomic and gene expression studies.

## Methods

### Gene expression dataset

To address the effects of copper on differential gene expression and alternative splicing, a gene expression dataset was acquired from our recent experiment investigating transcriptional consequences of acute copper exposure in *D. pulex* [53] (study accession PRJEB28650 in the ENA database). This transcriptomic dataset consisted of a total of 6 control samples and 15 copper exposed samples from three different clonal lineages (Fig. 1a). Briefly, one *D. pulex* individual was isolated from each of three habitats varying in historical copper contamination levels and cultured in the laboratory for about a year to establish the three clonal lineages (D, S and K). Toxicity assays were conducted in the lab to evaluate the relative Cu tolerance of each clone. Two clones (D and S) are from habitats not known to be contaminated with heavy metals, whereas the other clone (K) is from a lake contaminated with high levels of Cu (100 μg/L) as well as Ni and Mn [53, 71]. The clones were subjected to an acute Cu exposure experiment for gene expression analysis, in which five replicates of each clone were separated into different tanks and either exposed to 90 μg/L Cu for 24 h or raised in a controlled environment without Cu. Experiments were conducted in FLAMES medium [72] at 18 degrees Celsius under a 16:8 light-dark cycle, and were fed 20,000 cells of algae per mL [53]. After the exposure experiment, two replicates from each clone in control conditions were selected for RNA extraction, as well as five replicates from each clone in Cu conditions (Fig. 1a). Total RNA was extracted from whole primiparous adult *Daphnia*, resulting in 21 RNA-seq libraries that were sequenced on two lanes of an Illumina HiSeq 2000 (100 bp paired-end reads) at Genome Quebec.

### Bioinformatics

The raw sequence data of each of the 21 samples were checked for sequencing quality using FastQC [73]. Low quality sequences were trimmed using Trimmomatic (ILLUMINACLIP:TruSeq3-PE.fa:2:30:15:8:true TRAILING:30 MINLEN:90 CROP:90) [74]. All sequences were trimmed to an equal length of 90 bp. Using STAR [75], the sequences were then mapped to a recently revised *D. pulex* reference genome (assembly PA42 3.0) [42]. The resulting BAM files along with the reference annotation file obtained from [42] were submitted to featureCounts [76] to determine raw read counts per gene. Differential expression analysis was performed between all the copper-exposed (*n* = 15) and control samples (*n* = 6) using DESeq2 v1.22.2 [77] and edgeR v3.24.3 [78]. The genes were considered to be differentially expressed (DE) between the two conditions if the False Discovery Rate (FDR) adjusted *p*-value was less than 0.05 in both DESeq2 and edgeR analyses. A reciprocal BLAST approach was used with default parameters in BLASTn [79] to determine if the DE genes identified in this study matched those identified in our previous study as reported in Chain et al. [53], in which we used the same transcriptome data but a different reference genome and annotation [41]. For each gene, only BLAST hits that had an E-value <1e-10 were considered, and the two top BLAST hits were considered if they were within 10-bit scores of one another.

Identification of alternative splicing (AS) events and differential splicing (DS) analysis was conducted using rMATS v3.2.5 [25], which uses reads spanning both splice junctions and exons. The splice junctions for all identified alternative splicing events were required to be supported by at least five uniquely mapped reads and additionally have a minimum of 10 nt overhang for A3SS, A5SS, ES and MXE in at least one sample in each condition. The genes were considered to be differentially spliced (DS) if the adjusted *p*-value after Benjamini-Hochberg correction was less than 0.05 and if the difference in exon inclusion levels (Δ|ψ|) was greater than 20%. To determine whether the clonal lineages respond differently to Cu stress, differential gene expression and differential splicing analysis was also carried out between the copper-exposed (*n* = 5) and control samples (*n* = 2) separately for each clone. While clone-specific analyses have lower statistical power to detect global effects of copper-exposure due to lower sample sizes, they can reveal lineage-specific differences that would not be detected from the global analysis. Splicing plots were generated using ggsashimi [80].

To identify potential biological pathways involved in response to acute Cu exposure, Gene Ontology (GO) enrichment was performed for the DE and DS genes with topGO v2.30.1 [81] using the default algorithm (weight01). This tests for enrichment of particular functional categories among the list of DS genes compared to all expressed genes in our samples. GO terms with a weighted *p*-value less than 0.05 were considered to be significantly enriched. The sequences of alternatively spliced exons were also used in searches against the NCBI conserved domains database to identify putative protein domains involved in alternative splicing.

## Supplementary information

**Additional file 1: Table S1.** Quality and mapping information for the 21 *Daphnia pulex* RNA-sequencing libraries against both the JGI V1.0 2011 assembly from Ensembl (as reported in Table S3 in Chain et al. 2019 [53]) and also against the newer Daphnia PA42 3.0 reference assembly from Ye et al. 2017 [42]. Sample names consist of tank number (T), individual ID number, and clone (D, K or S). Samples were run in one of two lanes, and average quality (Q) is presented as PHRED scores. The % mapped reads and number of expressed genes are shown for each of the reference assemblies, as well as the alternative spliced genes against the newer reference. **Table S2.** Differentially expressed genes in response to acute Cu exposure when comparing all 6 controls with all 15 Cu exposed samples. **Table S3.** Summary of blast analysis comparing the differentially expressed (DE) genes identified by Chain et al. [53] with the genes from the newer Daphnia PA42 3.0 reference assembly from Ye et al. 2017 [42]. **Table S4.** Comparison of the DE genes identified by Chain et al. 2019 [53] and the genes from the newer Daphnia PA42 3.0 reference assembly from Ye et al. 2017 [42] using BLASTn. **Table S5.** Differentially spliced genes in response to acute Cu exposure when comparing all 6 controls with all 15 Cu exposed samples. Δ|Ψ| is the difference in the exon/intron inclusion level between the two conditions. **Table S6.** Differentially expressed genes in response to acute Cu exposure from clone specific analyses. **Table S7.** Genes commonly differentially expressed from global analysis and clone specific analyses. **Table S8.** Differentially spliced genes in response to acute Cu exposure in Clone D. **Table S9.** Differentially spliced genes in response to acute Cu exposure in Clone S. **Table S10.** Differentially spliced genes in response to acute Cu exposure in Clone K. **Table S11.** Gene Ontology enrichment analysis of the DE genes from the global analysis combining all 6 controls with all 15 Cu exposed samples (BP: biological process, MF: molecular function). **Table S12.** Gene Ontology enrichment analysis of the DE genes from clone specific analyses (BP: biological process, MF: molecular function). **Table S13.** Gene Ontology enrichment analysis of the DS genes from the global analysis combining all 6 controls with all 15 Cu exposed samples (BP: biological process, MF: molecular function). **Table S14.** Gene Ontology enrichment analysis of the DS genes from clone specific analyses (BP: biological process, MF: molecular function).

## Data Availability

The dataset used and analyzed in the current study is available from the ENA database under study accession PRJEB28650.

## Abbreviations

A3SS: Alternative 3′ splice site
A5SS: Alternative 5′ splice site
AS: Alternative splicing
Cu: Copper
DE: Differential expression
DS: Differential splicing
ES: Exon skipping
GO: Gene ontology
IR: Intron retention
mRNA: Messenger RNA
MXE: Mutually exclusive exon
PK: Pyruvate kinase
PSI: Percent spliced in
PTM: Post translational modification
